# Nutrition and Athlete Bone Health

**DOI:** 10.1007/s40279-019-01161-2

**Published:** 2019-11-13

**Authors:** Craig Sale, Kirsty Jayne Elliott-Sale

**Affiliations:** Musculoskeletal Physiology Research Group, Sport, Health and Performance Enhancement Research Centre, School of Science and Technology, Nottingham Trent University, Nottingham, NG11 8NS UK

## Abstract

Athletes should pay more attention to their bone health, whether this relates to their longer-term bone health (e.g. risk of osteopenia and osteoporosis) or their shorter-term risk of bony injuries. Perhaps the easiest way to do this would be to modify their training loads, although this advice rarely seems popular with coaches and athletes for obvious reasons. As such, other possibilities to support the athletes’ bone health need to be explored. Given that bone is a nutritionally modified tissue and diet has a significant influence on bone health across the lifespan, diet and nutritional composition seem like obvious candidates for manipulation. The nutritional requirements to support the skeleton during growth and development and during ageing are unlikely to be notably different between athletes and the general population, although there are some considerations of specific relevance, including energy availability, low carbohydrate availability, protein intake, vitamin D intake and dermal calcium and sodium losses. Energy availability is important for optimising bone health in the athlete, although normative energy balance targets are highly unrealistic for many athletes. The level of energy availability beyond which there is no negative effect for the bone needs to be established. On the balance of the available evidence it would seem unlikely that higher animal protein intakes, in the amounts recommended to athletes, are harmful to bone health, particularly with adequate calcium intake. Dermal calcium losses might be an important consideration for endurance athletes, particularly during long training sessions or events. In these situations, some consideration should be given to pre-exercise calcium feeding. The avoidance of vitamin D deficiency and insufficiency is important for the athlete to protect their bone health. There remains a lack of information relating to the longer-term effects of different dietary and nutritional practices on bone health in athletes, something that needs to be addressed before specific guidance can be provided.

## Key Points

**Table:** 

The diet required by the athlete to support bone health is not markedly different from the general population, with a few specific challenges.
An energy availability of 45 kcal kg of lean body mass (LBM)^−1^·day^−1^ is ideal to support bone health in the athlete, although this is an unrealistic target for many. Current knowledge would suggest trying to achieve an energy availability above 30 kcal·kgLBM^−1^·day^−1^ to minimise negative effects on the bone.
Athletes often consume 2–3 times more protein than recommended daily amounts, which is now thought to have no negative effects on bone health (and possibly beneficial effects), assuming adequate dietary calcium intake.
Dermal calcium loss might be an important consideration for some endurance athletes, who might wish to consider increasing calcium intake before exercise.
Much more athlete-specific research is required.

## Introduction

Bone mass [or bone mineral density (BMD)] changes across the lifespan and is characterised by a rapid phase of bone mass accrual during childhood and adolescence, a relatively quiescent stage during middle age, followed by age related bone loss during the latter years. The response of bone mass to ageing is similar in men and women, although men tend to attain a higher peak bone mass [1] and the age-associated losses of bone mass tend to be accelerated in women, particularly in the early post-menopausal period, when the protective effects of oestrogen on bone are withdrawn [2]. As such, women tend to be more susceptible to a clinically relevant degree of bone loss, culminating in the development of osteopenia and/or osteoporosis [3], although these are most definitely not exclusively female conditions.

Osteoporosis is defined, by the World Health Organization [4], as “a progressive systemic skeletal disease characterised by low bone mass and micro-architectural deterioration of bone tissue, with a consequent increase in bone fragility and susceptibility to fracture”. Consequently, bone mass and strength are important considerations in the prevention of osteoporosis and its associated conditions. Having low bone mass in itself is not necessarily a major clinical problem; the issues arise from the associated increase in bone fragility and heightened risk of osteoporotic fracture. Whilst there are no comprehensive data that are specific to the athlete population, osteoporosis is a common disease in the general population, which is estimated to affect 22 million women and 5.5 million men in the EU [5]. As might be expected, given these statistics, the rates of osteoporotic fracture are also high across Europe, with 620,000 hip fractures, 520,000 vertebral fractures, 560,000 forearm fractures and 1,800,000 other fractures being reported in 2010 [5]. Most importantly, it is clear that osteoporosis can significantly affect one’s quality and quantity of life, given that around 50% of hip fracture patients do not return to independent living and one fifth of individuals requiring hospitalisation for fragility fractures die within a 6-month period [6]. As such, the potential for development of such a bone condition in athletes requires careful consideration.

There are two main considerations for athletes concerning their bone health. Firstly, 90% of peak bone mass is achieved by the age of 20 years and the amount of bone accrued by the age of 30 years is about the maximum amount that will be attained [7, 8]. Secondly, it is very difficult to generate a sufficient and sustained osteogenic stimulus to improve bone health to such a degree as to offset age-associated bone loss. As such, it is important for athletes to maximise and protect their bone health during their athletic career, rather than sacrificing this for their athletic performance. Often, younger athletes are more concerned with their current performance level than the potential future risk to their health; in particular, there is a misconception that athletes can fully regain bone mass and strength once they have retired from sport. There are also potential performance consequences of poor bone health, such as the development of stress fracture injuries. These are important injuries for the athlete that can result in a significant loss of training time [9], which undoubtedly impacts upon sporting performance.

In general, exercise across the lifespan is considered beneficial for bone strength, as well as for many other associated aspects of ageing well [1]. Certainly, there is evidence to support this from some athlete groups that have stronger bones, particularly in sports involving high-impact forces and multi-directional movements (e.g. rugby, football, volleyball, hockey and combat sports). Conversely, not all sports have been shown to exert a positive influence on bone, with some athletes competing in sports, such as distance running, road cycling and swimming, having lower bone mass than their counterparts in other sports, controls or reference populations (for a review of this topic, please see Scofield and Hecht [10]). In addition, jockeys [11, 12] and ballet dancers [13, 14] are examples of athletes participating in sporting activities who have lower bone mass at some skeletal sites. In general, these sports tend to be characterised as being non-weight bearing, endurance based and/or are associated with low energy availability. A point worthy of further consideration is how best to evaluate bone health in the athlete and whether it makes sense or not to compare athlete data to normal population data, such as the T score that compares how much one’s bone mass deviates from the bone mass of the average healthy 30-year-old adult or the Z score that compares average bone mass to people of the same age and sex. Given that many athletes would be considered smaller (e.g. marathon runner, jockey) or larger (e.g. rugby prop forward) than the average individual, one might question whether estimating an athlete’s bone health by dual energy x-ray absorptiometry (DXA) T or Z score might be slightly misleading.

It is important that athletes consider the implications of their sport on their long-term bone health (e.g. risk of osteopenia and osteoporosis), as well as the risk of developing bony injuries (including medial tibial stress syndrome and stress fractures) in the shorter term. In order to develop potential strategies to support the athlete in this endeavour, consideration could be given to the factors that influence bone strength/weakening. Some of these factors, such as genetics, race, age and sex are non-modifiable; but some lifestyle factors provide a potential modifiable effect on the bone. Of these factors, mechanical loading has arguably the greatest effect; bone responds to the magnitude, rate, total number and direction of loading cycles induced through activity, which is described by Frost’s mechanostat theory [15]. As such, manipulating the mode, duration and intensity of exercise could be useful ways to improve bone health in athletes. This would require some manipulation of training schedules, and whilst there might often be scope to do this, that sort of advice rarely proves popular with coaches and athletes. As such, there is a need to consider other modifiable options, such as diet and nutrition.

The effects of dietary intake on bone health across the lifespan have been the subject of several recent narrative reviews, which we will draw the reader’s attention to, but not replicate. The purpose of this narrative review is to provide an overview of the potential dietary and nutritional influences on bone health, with a specific focus on the athlete.

## Nutrition and Bone Health

Bone is a nutritionally modulated tissue, which is evidenced by the acute reduction in bone metabolic markers that occurs with feeding in postmenopausal women [16]. Reductions, by up to 50%, in markers of bone resorption have been shown following feeding with, for example, carbohydrate, fat, protein, and calcium (for a comprehensive review, see Walsh and Henriksen [17]). Reductions in markers of bone formation also occur with nutrient feeding, although to a lower magnitude than for bone resorption markers [17]. Babraj and colleagues [18] have also shown that intravenous ‘feeding’ of glucose, lipid emulsion and amino acids (in the ratio of 55%:30%:15% energy) in healthy young men increased bone collagen synthesis by around 66%. In addition to modulating the daily rhythm of bone turnover [19], feeding can also moderate a number of hormones (such as calcitropic hormones, incretin hormones, growth hormone and cortisol) that are implicated in bone turnover in healthy postmenopausal women. Coverage of these responses is beyond the scope of the current review, but a detailed review is provided by Walsh and Henriksen [17].

What is clear is that nutrition has a significant influence on bone health across the lifespan, and this is well covered in the narrative review by Mitchell et al. [20]. In the main, the nutritional requirements to support the skeleton during growth and development and during ageing (Table 1) are unlikely to be notably different between athletes and the general population. As such, key nutrients for bone health, namely calcium, protein, magnesium, phosphorus, vitamin D, potassium and fluoride [21], should be considered as vital constituents of the athletes’ diet in order to directly support bone formation. In addition to these nutrients, the athlete should also ensure adequate intake of silicon [22], manganese, copper, boron, iron, zinc, vitamin A, vitamin K, vitamin C and the B vitamins [21, 23], in order to support other metabolic processes important for bone health. It is difficult to be specific on the recommended dietary intakes of particular nutrients for the athlete given that different countries have different recommendations for these intakes (for examples, see the guidelines from the European Food Safety Authority, National Health and Medical Research Council and the Institute of Medicine). What is also unclear is whether the hard training undertaken by athletes modifies these requirements for many of the nutrients relevant to bone health. Of course, the majority of recommended dietary intake guidelines consider the potential for variation to allow them to meet the needs of the majority of the population, but many of these guidelines are focused upon preventing nutrient deficiencies, whereas the athlete is more focused upon supporting optimal function (a useful resource here is Larson-Meyer et al. [24]). As such, there might be a need for the athlete to consider a well conducted nutritional assessment of their dietary intake to identify whether or not they are consuming the required amounts of the key nutrients to underpin bone health, among other things, including optimal performance. In terms of foods, most recommendations for good bone health include the consumption of dairy, fish, fruits and vegetables (particularly of the green leafy kind), which are useful sources of the main nutrients supporting bone health. When the intake of particular nutrients of benefit to bone is difficult (perhaps because of food intolerances or food preferences), some consideration could be given to the consumption of fortified foods or supplements.

**Table 1:** Some key nutrients to support bone health

Nutrient	Role in bone	Some possible sources
Protein	Part of the organic matrix of bone for collagen structure. Has a role in the production of hormones and growth factors that modulate bone synthesis. Protein might have an indirect effect on the bone through its support for muscle mass and function, but also via the increase in circulating levels of IGF-1, which has an anabolic effect on bone	Meats, dairy (milk, yoghurt, cheese), eggs, fish, nuts, beans, pulses
Calcium	A major bone forming mineral. 99% of the body’s calcium is stored in the bone. Conversely, low calcium levels in the diet can contribute to a catabolic effect on the bone through the activation of PTH	Dairy (milk, cheese, and yogurt), spinach, kale, okra, collards, soybeans, white beans
Phosphorus	Phosphorus plays an integral role in bone formation as it is an essential constituent for the mineralisation of bone, and low phosphorus levels contribute to an impairment in bone mineralisation. Equally, there are issues with diets that are very high in phosphorus, particularly if combined with a low intake of dietary calcium, which can lead to increased PTH and indicatively a catabolic effect on bone	Dairy (milk, yoghurt), meats, poultry, fish, nuts, beans
Vitamin D	An important direct and/or indirect mediator of bone, that is certainly important for intestinal calcium and phosphorus absorption via 1,25(OH)2D stimulation, which is subsequently related to PTH secretion and activity	Fatty fish (tuna, mackerel, salmon), cheese, egg yolks, fortified foods
Magnesium	More than half of the body’s store of magnesium is in the bone, and it plays an important role in organic matrix bone synthesis. The controlled regulation of magnesium homeostasis is suggested to be important for bone health, due to the fact that there might be harmful effects of both a deficiency and an excess of magnesium. Magnesium deficiency contributes directly to poor bone health (due to its importance for both osteoblasts and osteoclasts) and indirectly by impacting on vitamin D and calcium to influence PTH secretion and activity. Conversely, high magnesium levels have also been associated with bone mineralisation defects	Whole grains, spinach, nuts (almonds, cashews, peanuts), quinoa, avocado, dairy
Zinc	Plays an important role in the mineralisation of bone tissue and organic matrix bone synthesis; as such, zinc status can be directly linked to bone turnover. Might also be important for the physiological action of vitamin D on calcium, thus potentially also indirectly influencing PTH secretion	Meats, shellfish, nuts, seeds, legumes
Copper	Its direct physiological action on bone is not as clear as for some other nutrients, although it is needed for enzyme activity to increase the cross-linking of collagen and elastin molecules. There is some suggestion that bone mineralisation might be affected in those with low copper intakes	Nuts, shellfish, offal
Boron	The physiological action of boron on bone remains unclear, although indirect effects through actions on vitamin D and oestrogen and through improved calcium and magnesium retention by the kidneys are possible	Fruits (raisins, prunes), nuts (almonds, hazelnuts, brazil nuts, walnuts, cashews), beans, lentils, wine
Manganese	Deficiency has been associated with reduced bone mass, potentially due to its role in the formation of bone regulatory hormones and some enzymes involved in bone metabolism	Tea, bread and cereals, nuts, green vegetables
Potassium	High potassium intakes have been associated with increased bone mass. Much of the effect of potassium on bone might be indirect and due to the protection provided against a high acid load that can influence the resorption of bone to release calcium. Indeed, the intake of potassium salts has been shown to reduce bone resorption and urinary calcium excretion	Bananas, broccoli, parsnips, Brussels sprouts, nuts and seeds, fish and shellfish, meats
Iron	Has important roles in vitamin D metabolism and collagen synthesis. Those with disorders of iron metabolism have been suggested to have lower bone mass and an increased risk of suffering an osteoporotic bone fracture. Interestingly, a very high intake of iron might also be bad for the bone, most probably due to the increased oxidative stress and inflammatory response	Liver (not during pregnancy), meats, beans, nuts, whole grains, dried fruits, green leafy vegetables
Vitamin K	Low intakes have been associated with osteopenia and increased fracture risk. Physiologically vitamin K has also been linked to under-carboxylation of osteocalcin, whereas supplementation with vitamin K might reduce bone turnover and improve bone strength	Green leafy vegetables, vegetable oils, cereal grains
Vitamin C	Vitamin C deficiency leading to scurvy has long been reported to result in bone pain. Vitamin C is important for collagen synthesis and is also a known antioxidant, which might explain both direct and indirect effects on the bone	Fruits (oranges, orange juice, strawberries, blackcurrants), peppers, broccoli, Brussels sprouts, potatoes
Vitamin A	Perhaps one of the more controversial nutrients with regards to a link to bone. There are suggestions that a high dietary intake of vitamin A is associated with a greater risk of osteoporosis and hip fracture. Conversely, intakes of some of the carotenoids, which are precursors of vitamin A, have been associated with higher bone mass. More research is required to determine optimal intakes of vitamin A for bone health	Liver and liver products (not during pregnancy), dairy (cheese, milk, yoghurt), eggs, oily fish
B Vitamins	An association between the intakes of vitamins B_2_, B_6_, folate and B_12_ and a reduction in the risk of osteoporosis and associated hip fracture has been suggested. Similarly, lower intakes of the B vitamins have been shown in patients with hip fracture. Mechanistic explanations for a link between the B vitamins and the bone would include a positive effect on collagen cross-link formation and increased bone resorptive activity when vitamin B deficient	Dairy (milk, cheese), eggs, fish, fresh and dried fruits, meats, vegetables
Silica	Deficiency is associated with poor skeletal development, probably due to its importance in the initiation of bone mineralisation, although its physiological role here is still poorly understood	Bananas, beer, green beans, bread, rice, carrots, cereals

For further information on recommended amounts and sources, the reader could refer to the following: Europe: European Food Safety Authority; Australia and New Zealand: National Health and Medical Research Council; USA: Institute of Medicine

*IGF-1* insulin-like growth factor 1, *PTH* parathyroid hormone

## Specific Nutritional Issues for the Athlete

Whilst, as mentioned above, many of the dietary requirements to support bone health in the athlete are likely to be largely the same as those supporting bone health in the general population, there are some dietary/nutritional challenges specific to the athlete. The remainder of this review will focus upon what we consider to be the most pertinent, namely: energy availability, low carbohydrate availability, protein intake, vitamin D intake and dermal calcium and sodium losses. The review will also briefly cover the effects of feeding around exercise on bone metabolism.

### Energy Availability

Energy availability can be described as the amount of ingested energy remaining to support basic bodily functions and physiological processes, including growth, immune function, locomotion, and thermoregulation, once the energy needed for exercise has been utilised [25]. For a good overview of the myriad effects of low energy availability in the athlete, we direct the reader to the recent review by Logue et al. [26]; herein, we focus specifically on the potential effects of low energy availability on the bone. One of the major problems of identifying those athlete populations at risk of low energy availability and of identifying the causal links between low energy availability and bone health is the significant difficulty in collecting accurate data on energy intake and energy expenditure (particularly during more intermittent types of exercise) [27].

The low energy availabilities experienced by some athletes can have adverse effects on bone [28], including acute bony injuries and longer-term reduced bone mass and strength. It seems that many highly active individuals, particularly elite and recreational endurance athletes, might have some difficulties in matching their dietary energy intakes to their exercise energy expenditure, which inevitably results in low energy availability [29, 30]. It is clear that this is also an issue that can affect male athletes as well as female athletes [31].

Ihle and Loucks [32] were among the first to directly investigate the effects of low energy availability on bone metabolism in healthy young women by inducing three reduced energy availabilities [compared to an energy balanced control at 45 kcal·kg of lean body mass (LBM)^−1^·day^−1^], 30, 20 and 10 kcal·kgLBM^−1^·day^−1^, in an independent-groups design, using both dietary manipulation and exercise. Whilst more moderate restrictions of energy availability (20 and 30 kcal·kgLBM^−1^·day^−1^) modulated bone formation [as determined by osteocalcin (OC) and carboxy-terminal propeptide of type 1 procollagen (P1CP) concentrations], no effect was shown upon bone resorption (as determined by N-terminal telopeptide [NTX] concentrations). OC concentrations were reduced by − 0.9 ± 0.3 ng·mL^−1^ at 30 kcal·kgLBM^−1^·day^−1^ and − 2.4 ± 0.5 ng·mL^−1^ at 20 kcal·kgLBM^−1^·day^−1^, and P1CP concentrations were reduced by − 16 ± 8 ng·mL^−1^ at 30 kcal·kgLBM^−1^·day^−1^ and − 28 ± 8 ng·mL^−1^ at 20 kcal·kgLBM^−1^·day^−1^. More severe reductions in energy availability (at 10 kcal·kgLBM^−1^·day^−1^) produced the dual effect of reducing bone formation (OC − 2.3 ± 0.5 ng·mL^−1^; P1CP − 48 ± 13 ng·mL^−1^) and increasing bone resorption (NTX +17 ± 4 nM BCE/mM Cr). Although the relevance of some of these markers of bone metabolism was questioned (they would not be considered the optimal markers of bone resorption and formation to use today [33]), this paper has been instrumental in raising the awareness of potential problems for the bone when energy availability is low.

It is common for athletes to experience low energy availabilities of a similar order of magnitude to those used by Ihle and Loucks [32]. Indeed, Thong et al. [34] reported that amenorrhoeic athletes have energy availabilities of ~ 16 kcal·kgLBM^−1^·day^−1^, which formed part of the rationale for the recent studies conducted by our research group [35, 36]. In the first of these studies [35], reducing energy availability to 15 kcal·kgLBM^−1^·day^−1^ over 5 days resulted in decreased bone formation [as determined by total procollagen type 1 N-terminal propeptide (P1NP)] and increased bone resorption [as determined by C-terminal telopeptide (β-CTX)] in women, but not in men. Despite this, examination of the individual data showed that some men responded to lower energy availability with a decrease in bone formation. Whilst this is in no way conclusive, there is the possibility that lower energy availability will affect bone metabolism by decreasing bone formation in men, but that it might take a lower level of energy availability to produce this response than in women. This would be an interesting avenue for future research.

One of the issues with examining the effects of reduced energy availability on bone metabolism in athletes and athletic populations in the laboratory is that this is usually achieved via a reduction in dietary intake and an increase in exercise energy expenditure. Whilst this is probably relevant, it does not allow us to determine whether the effect of low energy availability on bone might be more as a result of dietary restriction or as a result of high exercise energy expenditures (or whether this makes no difference). Recently, we have examined the effects of 3 days of low energy availability, again at 15 kcal·kgLBM^−1^·day^−1^, achieved by either diet or exercise, on bone turnover markers in active, eumenorrhoeic women [36]. Low energy availability achieved through dietary energy restriction resulted in decreased bone formation, with no concomitant change in bone resorption. Low energy availability achieved through exercise alone, on the other hand, did not significantly alter bone metabolism. Taken together, these results might suggest some bone protective effect of the mechanical loading induced by exercise in the short term, even when this might result in low energy availability. These results also suggest that the athlete must focus on adequate dietary intake during hard training periods.

Given the potential for low energy availability to negatively influence the short-term responses of bone, it would seem sensible to suggest that if this state was maintained over longer periods, more serious consequences might be experienced. This raises an important, but as yet unanswered, question over whether it is the magnitude of the low energy availability (i.e. there is a threshold below which there is a negative effect on the bone) that is important or whether it is more an issue of continuous low energy availability over time that negatively influences bone health. Certainly, it would seem that bone metabolism recovers relatively quickly from short-term low energy availability [37], whilst several cross-sectional studies have shown that those maintaining low energy availability over time have lower bone mass, poorer bone structure and/or an altered bone metabolic profile compared with those who do not experience low energy availability [11, 38–41]. Added to this is the evidence from the many studies conducted since 2007 relating to the female athlete triad [25, 42]. More recently, this same group has also suggested the potential for a similar syndrome in male athletes (referred to as the male athlete triad; see Tenforde et al. [43]), which mirrors the suggestions made relating to the occurrence of impaired bone health as a result of low energy availability, by the relative energy deficiency in sport (RED-S) paradigm [44, 45]. Whilst further discussion of these conditions (the male athlete triad and RED-S) is vitally important and would be highly relevant herein, these topics are covered more extensively in another article within this supplement.

Whilst it might seem sensible to suggest to the athlete that maintaining an energy availability of 45 kcal·kgLBM^−1^·day^−1^ over time is necessary to optimise their bone health and protect against bony injuries, it is probably an unrealistic target for many athletes. Certainly, it seems unlikely that elite endurance athletes (male or female) would be able to attain these levels of energy availability given the high energy expenditures induced by training and the limited time for refuelling that their demanding training schedules allow. Another complication here is that endurance athletes might be directly opposed to trying to maintain a balanced energy intake, since many consider an energy deficit as essential to drive the endurance phenotype. Taken together, these points highlight the difficulty in maintaining balanced energy availabilities for the promotion of bone health in the endurance athlete when stacked against the competing interests of optimising their sporting performance. As such, further research is needed to identify whether or not there is a means to maintain bone health without compromising training practices to optimise endurance performance. One possibility might be to periodise low energy availability into the training cycle to develop the endurance phenotype without the need to have constantly low energy availability, a recent approach suggested by Stellingwerff [46].

Further research is also required to tease out the nuances of the effects of energy and nutrient availability on bone. In the laboratory, energy intake is often limited by simply determining habitual dietary energy intake and then cutting this intake down by a certain percentage. The issue with this is that nutrient intake is also reduced by the same relative amount, which begs the question of whether the effects on bone are wholly energy availability dependent or whether the concomitant reduction in the availability of carbohydrate, protein, calcium, vitamin D and other micronutrients also contributes to the negative impact on bone. In addition, there might also be an interaction between elements of the female athlete triad and certain nutrients that could exacerbate the effects on bone. For example, iron deficiency might directly interact with reduced energy availability to further disrupt thyroid function and to suppress anabolic factors for bone formation, as recently postulated by Petkus et al. [47].

### Low Carbohydrate Availability

There is evidence to suggest that some athletes (particularly endurance athletes) might benefit from either lower carbohydrate diets or low-carbohydrate/high-fat diets in terms of their performance, in addition to the proposed benefits for body composition [48, 49]. This, however, remains a matter of some contention, given that historically carbohydrate intake would provide the largest contribution to energy intake in the athlete’s diet and that low-carbohydrate diets could present a risk for a low energy availability state. Whilst no studies have directly examined the effects of low carbohydrate availability on bone health parameters in athletes, it has been shown that carbohydrate feeding can reduce bone turnover [50]. Bjarnason et al. [50] reported a reduction of around 50% in bone resorption marker (β-CTX) concentrations following the administration of a standard 75-g oral glucose tolerance test. Similarly, the provision of carbohydrate has been shown to attenuate the bone resorption response to acute exercise in athletes involved in an 8-day overloaded endurance training trial [51]. Sale et al. [52] also showed a modest post-exercise reduction in PINP and β-CTX with carbohydrate feeding immediately before, during and immediately after a 120-min treadmill run in recreationally active individuals.

There is some more direct information to suggest that following a low-carbohydrate diet would negatively affect bone health, albeit from animal models and when concomitantly followed with a high-fat diet [53]. Bielohuby et al. [53] measured bone growth, BMD and bone turnover in growing rats fed for 4 weeks on either normal chow (9% fat, 33% protein, and 58% carbohydrates) or on two different low-carbohydrate/high-fat diets (1: 66% fat, 33% protein, and 1% carbohydrates; 2: 94.5% fat, 4.2% protein, and 1.3% carbohydrates). They showed that longitudinal growth, BMD, and bone mechanical properties were all impaired by both low-carbohydrate/high-fat diets, which they suggested was potentially mediated by the reductions in insulin-like growth factor 1 (IGF-1) levels shown. Bone formation markers and the expression of transcription factors influencing osteoblastogenesis were also reduced on the low-carbohydrate/high-fat diets, which the authors suggested might indicate a lower rate of mesenchymal stem cell differentiation to osteoblasts. Conversely, in humans, albeit osteoarthritis patients and not athletes, there was no effect on bone turnover (as assessed by urinary N-telopeptide and bone-specific alkaline phosphatase concentrations) when patients were fed less than 20 g of carbohydrate per day for 1 month and then less than 40 g of carbohydrate per day for the next 2 months [54].

As such, there might be some suggestion that following a low-carbohydrate diet acutely, chronically or even periodically might negatively influence the athlete’s bone health, but this is by no means certain. Future research work is required to determine whether low-carbohydrate dietary practices would negatively impact the bone health of athletes in the longer term.

### Protein Intake

Athletes are often recommended to consume more protein than is recommended for the general population, in order to support the additional demands of athletic training. The recommendations for athletes is to consume between 1.2 and 1.6 g·kgbw·day^−1^, although under certain circumstances this recommendation might increase to 2.2 g·kgbw·day^−1^ [55], which is higher than the 0.8 g·kgbw·day^−1^ recommended to the general population. This may result in a conflict of interest, as there is a long-held belief that higher protein intakes may have a negative influence on bone health [56, 57], a topic that has recently been covered in detail by Dolan and Sale [58]; herein we will summarise the salient points. The ‘acid-ash hypothesis’ [59] suggests that animal proteins are acidic (essentially having a high potential renal acid load) and, as such, provide a significant challenge to the maintenance of acid–base balance by disrupting the body’s pH, which is critical to the maintenance of homeostasis. The theory suggests that, in order to protect the homeostatic state, the body increases the availability of alkaline minerals, such as calcium, most of which are stored within the bone tissue. Indeed, around 99% of the calcium stored within the body is stored within the bone and so any requirement for the release of calcium into the circulation to counteract the effects of increased acidity is likely to result in the resorption of bone [59]. The calcium released from the bone in order to counteract a high potential renal acid load is also associated with increased losses of calcium in the urine, along with lower BMD and an increased rate of bone loss [60]. Taken together, the results of these studies would suggest that, as a result of the acid-ash hypothesis, an athlete consuming a high (particularly animal) protein diet would run the risk of inducing demineralisation of the bone over the longer term with potential adverse effects on bone health.

Taken alone, however, this theory does not provide a fully balanced account of the potential influences of a high protein intake on bone. The main negative effect of a high animal protein diet on bone according to the acid-ash hypothesis relies upon the clear assumption that the calcium used to neutralise the high potential renal acid load resulting from animal protein consumption comes from the bone and that any excess calcium subsequently excreted in the urine comes from the bone. This might not, however, be the case given that Kerstetter et al. [61] have shown that higher protein intakes resulted in an increase in the amount of calcium that is absorbed from foods, and, as such, the increased urinary calcium levels with high animal protein intakes might well come from increased calcium availability instead. Of further consideration is the fact that dietary acid load could just as easily be influenced by a reduction in the intake of alkaline foods, such as fruits and vegetables, as by an increase in the intake of acidic foods, such as animal proteins. This would compound the issue, especially given that alkaline foods are also rich in a wide range of micro- and phyto-nutrients that are important for bone health [21]. Therefore, it is possible that the poorer bone outcomes reported in those consuming an acidic diet [60] were not due to high protein, but were as a result of a shortage of nutrient rich fruits and vegetables. This gives further support to the point made in Sect. 2 that athletes should consume fruits and particularly green leafy vegetables to support their bone health.

It is equally important to consider the possibility that protein is, in fact, beneficial and not harmful to bone (for a review, see Dolan and Sale [58]). Bone tissue is ~ 50% protein by volume and about a third by mass [62], given that it is an important constituent of the structural matrix of bone [63]. As such, athletes need to consume sufficient protein to support the increased rate of bone turnover caused by athletic training. Additionally, protein ingestion increases the production of a number of hormones and growth factors, such as IGF-1, which are also involved in the formation of bone. Of further relevance for the athlete is the fact that higher protein intakes also support the development of muscle mass and function [64]; the associated increases in muscular force would likely act upon the bone to enhance bone mass and strength [65].

On the balance of the available evidence it would seem unlikely that higher animal protein intakes, in the amounts recommended to athletes, are harmful to bone health. This is evidenced by the results of a number of studies (albeit not in athletes per se) that have been well summarised and statistically combined in high-quality meta-analyses (as summarised by Rizzoli et al. [66]). It might, however, be sensible to recommend to athletes that they maintain adequate calcium during periods of higher protein consumption to be sure of no negative effects on the bone. A small positive effect of protein on BMD and fracture risk has been identified, suggesting that the protein intakes of athletes, which are usually in excess of the recommended daily allowance, might be ultimately beneficial to the bone, although this requires further specific research.

### Vitamin D Intake

Numerous studies in the last 5–10 years have identified athlete groups who have deficient or insufficient levels of circulating vitamin D [67], although the specific definitions of vitamin D deficiency and insufficiency have been debated. Whilst there is broad agreement that vitamin D deficiency is defined as a serum 25-hydroxyvitamin D [25(OH)D] level below 25 nmol·L^−1^ [68, 69], there is no consensus as to what constitutes insufficiency or indeed the optimal vitamin D status. The Institute of Medicine Report [70] on dietary reference intakes for vitamin D suggested that 25(OH)D levels of 40 nmol·L^−1^ were sufficient to cover the requirements of ~ 50% of the US population, with levels of 50 nmol·L^−1^ being enough to cover the requirements of at least 97.5% of the US population.

Given the well-identified link between low vitamin D levels (serum 25-hydroxyvitamin D [25OHD] levels below 25 nmol·L^−1^) and bone, where it plays an important role in calcium and phosphorus regulation in the body, it is highly likely that athletes who are deficient in vitamin D would be at a greater risk of low bone mass and bone injuries [71], such as stress fractures.

Whilst the causes of vitamin D deficiency in the general population are clearly multifactorial, it is most likely that the main cause in the athletic population is a reduction of ultraviolet B radiation absorption into the skin, which is the major source of vitamin D [72, 73]. Whilst this seems fairly obvious in relation to those athletes who largely train and compete indoors and those who live and train in latitudes furthest from the equator, it might also be of relevance to those who train and compete outside, but who have to wear a significant amount of equipment (e.g. jockeys) or who choose to use high sun protection factor sunscreen or sunblock (which rightly relates to considerations over the protection of the athlete’s skin from damage).

A direct relationship between serum vitamin D levels and musculoskeletal outcomes is relatively clear [69] and makes sense given the important role for vitamin D in calcium and phosphorus metabolism. Miller et al. [74] examined the vitamin D concentrations in 53 patients with radiographically confirmed stress fractures, with 44 of these patients having serum vitamin D levels of less than 40 ng·mL^−1^. Similarly, Maroon et al. [75] showed that vitamin D levels were significantly lower in professional American Football players having suffered at least one bone fracture when compared to those players with no fractures. Conversely, female Navy recruits receiving 2000 mg calcium plus 800 IU of vitamin D per day had a 20% lower incidence of stress fracture than the recruits receiving the placebo [76]. Whilst not directly causal, low-fat dairy products and the major nutrients in milk (calcium, vitamin D, and protein) were associated with greater bone gains and lower stress fracture rates in young female runners [77]. Interestingly, a higher potassium intake was also associated with greater gains in hip and whole-body BMD.

It would seem relatively clear that the avoidance of vitamin D deficiency and insufficiency is important for the athlete to protect their bone health. In theory, this is relatively straight forward, and achieving serum vitamin D levels, through dietary supplementation, above 50 nmol·L^−1^ would most likely prove protective [78], although a clear target for vitamin D in the prevention of bone injury prevention remains unknown.

### Dermal Calcium and Sodium Losses

Athletes who undertake a high volume of prolonged exercise, particularly when that exercise is not weight bearing, are at risk of having lower BMDs [79, 80]. One of the potential contributors to this might be an increase in bone resorption mediated by the activation of parathyroid hormone due to reductions in serum calcium levels, which, in turn, occur as the result of dermal calcium losses [81]. It is likely that the level of dermal calcium loss required to cause a decline in serum calcium concentrations, which is sufficient to activate parathyroid hormone secretion and thus bone demineralisation, would only occur during prolonged hard exercise. Given that calcium plays an important role in many cellular processes that occur while exercising, the body vigorously defends serum calcium concentrations, predominantly by the demineralisation of bone, which, in turn could lead to a reduction in bone mass over time. As such, Barry et al. [81] proposed that supplementing with calcium before or during exercise might compensate for dermal calcium losses and defend the serum calcium level, meaning that there would be no concomitant increase in parathyroid hormone release or bone resorption.

Barry et al. [81] examined whether calcium supplementation, either before or during cycling exercise, reduced the exercise-induced increases in parathyroid hormone and bone resorption (as determined by β-CTX). Twenty male endurance athletes completed a 35-km cycling time trial on three occasions having consumed either (1) 1000 mg of calcium 20 min before exercise and a placebo during exercise; (2) a placebo before exercise and 250 mg of calcium every 15 min during exercise; or (3) a placebo before and during exercise. The results showed that when 1000 mg of calcium was ingested as a single bolus prior to exercise, there was an attenuated parathyroid hormone response to the subsequent exercise bout. There was a smaller attenuation of the parathyroid hormone response when calcium was supplemented during exercise, and this did not reach statistical significance.

Following on from this work, others have also shown that pre-exercise calcium consumption, this time in the form of a high-calcium meal (~ 1350 mg), attenuated the subsequent response of both parathyroid hormone and bone resorption (assessed via β-CTX concentrations) to a 90-min cycling bout in competitive female cyclists [82]. Although these results suggest that pre-exercise calcium consumption/supplementation may represent an optimal strategy for preventing bone resorption during exercise, the chronic effects of this nutritional strategy on BMD are yet to be researched. It is unlikely that the amount of calcium lost in the sweat would be significant enough to cause perturbations to calcium homeostasis to the extent that it would affect bone metabolism, unless the sweat rate was fairly high and/or the duration of sweat loss prolonged. As such, it is likely that this would be of primary concern for the endurance and ultra-endurance athlete, but perhaps also for any athlete who uses dehydration mechanisms to ‘make weight’. This latter possibility has not been explored and future research is required.

In line with this, there is also the possibility that the challenge to fluid and sodium homeostasis that would occur under these circumstances might influence bone metabolism and health. This, to our knowledge, has not been directly or well-studied in relation to the athlete, but there is some suggestion from the osteoporosis focussed literature suggesting that bone might be negatively affected by hyponatraemia. Verbalis et al. [83] examined the effects of using a syndrome of inappropriate antidiuretic hormone secretion rodent model to show that 3 months of hyponatraemia (~ 30% compared with normonatraemic controls) significantly reduced the BMD of excised femurs and reduced both trabecular and cortical bone, purportedly via an increase in bone resorption and a decrease in bone formation. The same paper also reported on a cross-sectional analysis of human adults from the Third National Health and Nutrition Examination Survey, showing that mild hyponatraemia was associated with significantly increased odds of osteoporosis, in line with the rodent data presented. This might be explained by novel sodium signalling mechanisms in osteoclasts resulting in the release of sodium from bone stores during prolonged hyponatraemia [84].

## Feeding and Acute Exercise

Nutrient ingestion around acute exercise can alter the bone resorption marker response to that exercise bout. Many athletes exercise in the morning after an overnight fast, which has the potential to promote an increase in bone turnover. Scott et al. [85] investigated the effects of feeding a mixed meal, versus fasting, on the bone metabolic response to a 60-min treadmill run at 65% of maximal oxygen uptake (VO_2max_) in physically active younger men. As anticipated, the ingestion of food reduced pre-exercise bone resorption (as measured by β-CTX), but, contrary to what was proposed, the bone resorption response to exercise was greater in the fed condition than in the fasted condition and, over time, there was no difference in the response between fasting and feeding. As such, it seemed that the mechanical loading induced by exercise might have provided a more powerful stimulus than that of pre-exercise feeding. In line with this theory, Sale et al. [52] examined the effect of feeding carbohydrate during exercise on the bone metabolic responses of physically active young men. Carbohydrate feeding attenuated bone resorption (β-CTX) and formation (P1NP) in the hours but not days following exercise, indicating an acute effect of carbohydrate feeding on bone turnover. These effects were, however, small and transient, perhaps reflecting the short timeframe and limited ability to feed during exercise (an 8% glucose solution was given immediately before and every 20 min during exercise, at a rate of 0.7 gCHO·kg^−1^BM·h^−1^). The total amount of glucose ingested was 102.1 ± 10.6 g in a total solution volume of 1276 ± 132 mL. Given the fact that the post-exercise period might provide a longer timeframe and a greater scope for intervention, Townsend et al. [86] investigated the influence of a combined carbohydrate/protein supplement following a treadmill run to exhaustion at 75% VO_2max_, in endurance trained young men. There were three trials conducted in this study: (1) placebo: ingested immediately and 2 h post-exercise; (2) immediate feeding: carbohydrate plus protein (1.5 g·kg^−1^BM dextrose and 0.5 g·kg^−1^BM whey protein) ingested immediately post-exercise and placebo ingested 2 h post-exercise; and (3) delayed feeding: placebo ingested immediately post-exercise and carbohydrate plus protein ingested 2 h post-exercise. When carbohydrate plus protein was ingested immediately post-exercise, there was a suppression of the exercise-induced bone resorption (β-CTX) response when compared to the control trial, along with a smaller increase in the bone formation (P1NP) response 3–4 h post-exercise.

It would seem clear that feeding around exercise can moderate the bone metabolic response to that exercise bout, with the post-exercise period being perhaps the most useful timeframe for intervention. Whilst the reduction in bone resorption might be expected to have a protective effect on the bone during periods of high-intensity and/or volume training, one must also consider the possibility that these responses are likely to be important in the adaptation of the bone. Longer-term studies are therefore required to determine whether or not these shorter-term or acute responses to feeding around exercise are positive for bone health. The studies in this area have largely been conducted in men, and it would be of interest to determine whether the same effects are seen in exercising women.

## Conclusion

Bone health is an important issue for some athletes, particularly those who are at a greater risk of low or lower BMD. These athletes should develop strategies to take care of their bones, particularly during adolescence and early adulthood, even at the expense of their training and performance, given that trying to overcome an already low bone mass in later life is extremely difficult. Taking care of their diet and nutrition might help athletes to better protect their bones against the demands of their sport. Dietary advice for athletes in this regard should remain in line with the advice given to the general population, with some consideration given to where there would be a need for higher intakes to match the needs of the sport and to optimise function, although there are several specific challenges that certain athletes might face over and above those faced by the general population. In this review, we have attempted to acknowledge some of these potential issues and highlight the information that is currently available to support these views. There is, however, a dearth of information relating to the effects (particularly the longer-term effects) of different dietary and nutritional practices on bone health in athletes, and significant research effort is required on this topic in the future. In terms of seeking the best possible practical advice, athletes could seek a well-qualified sports dietician/nutritionist.

### Some Suggestions for Future Work

- There is still a requirement to clearly define which types of athlete are and which types of athlete are not at risk of longer-term bone health issues, such as osteopenia and osteoporosis.
- Further research is needed to determine the wider implications of reduced energy availability, beyond bone, as suggested by the RED-S syndrome; currently these are not well researched.
- It remains to be clearly established whether there is or is not a male athlete triad and whether the bone health implications of reduced energy availability are seen at the same level as in females or whether males are a little more resistant to the effects of low energy availability.
- It remains to be determined whether there is or is not a threshold beyond which lower energy availabilities (less than 45 kcal·kgLBM^−1^·day^−1^) do not result in reduced bone formation.
- Further research is required into the periodisation of low energy availabilities in endurance athletes, such that they can benefit from the positive effects of calorie restriction on the endurance phenotype but without putting their bone health at risk.
- More work is required in athletes to determine the effects of nutrient availability (particularly of carbohydrate) separately from energy availability on bone health.
- Although there are data available to suggest that pre-exercise calcium consumption and/or supplementation can reduce the bone resorption response to endurance exercise, the chronic effects of this strategy on bone mass and strength are not yet known.
- The amounts of calcium lost during training in endurance and ultra-endurance athletes are still not well known, nor is the amount of calcium lost during more passive sweating, particularly in hot environments, such as might be performed by weight-making athletes.
- No research has been conducted in athletes to determine whether or not there is an effect of sweat sodium loss on bone.
- Longer-term studies are needed to determine whether or not the shorter-term or acute responses of bone metabolism to feeding are positive for bone health. These studies should also seek to determine whether feeding should be periodised around hard training blocks rather than constant so as not to reduce the potential adaptation of the bone to exercise training.
